# Human Intestinal Spirochetosis: A Rare Case of Intermittent Bloating and Hematochezia

**DOI:** 10.7759/cureus.25756

**Published:** 2022-06-08

**Authors:** Reinaldo L Pérez Moux, Pooja Mude, Shreyans Doshi, Kiran Madwani, Amanda Barrett, John Erikson L Yap

**Affiliations:** 1 Internal Medicine, Augusta University Medical Center, Augusta, USA; 2 Internal Medicine, Augusta University Medical College of Georgia, Augusta, USA; 3 Gastroenterology and Hepatology, Augusta University Medical College of Georgia, Augusta, USA; 4 Pathology, Augusta University Medical College of Georgia, Augusta, USA

**Keywords:** rare infectious diseases, screening colonoscopy, adult gastroenterology, spirochetes, hematochezia, abdominal bloating, hiv-patient, human intestinal spirochetosis

## Abstract

Human intestinal spirochetosis (HIS) is a condition where spirochetes, a group of spiral-shaped bacteria, attach to the apical membrane of the human colorectal epithelium. Although most findings of HIS are simply incidental discoveries found during screening colonoscopies, the ability to mimic the presentation of inflammatory bowel diseases should prompt consideration of this condition as part of a working differential diagnosis. Herein, we present the case of a 57-year-old bisexual, African American male with a medical history of Human Immunodeficiency Virus (HIV) on antiretroviral therapy (ART) with an undetectable viral load that presented for an elective, outpatient colonoscopy after experiencing four months of intermittent bloating and hematochezia. Histologic examination of colonic biopsies confirmed a diagnosis of HIS. The nonspecific clinical presentation in the setting of well-controlled HIV makes HIS a formidable diagnostic challenge that requires increased awareness.

## Introduction

Commonly linked to diarrheal illness in the veterinary world, intestinal spirochetosis (IS) is not as well understood as a human disease [1]. In human intestinal spirochetosis (HIS), spirochetes attach to the apical membrane of the colorectal epithelium and are generally non-invasive. These thin, spiral-shaped, highly mobile, gram-negative, double-membrane bacteria have been classified into three main families: Spirochaetaceae, Leptospiraceae, and Brachyspiraceae; the latter being more associated with the intestinal colonization, specifically the *Brachyspira aalborgi *and *Brachyspira pilosicoli* species [2]. Although the presence of the bacteria in the stool does not necessarily correlate with IS or its clinical symptoms, there is a noticeable prevalence of intestinal spirochetes in developing regions. In developed countries, only men who have sex with men and HIV-positive patients, regardless of the degree and extent of immunodeficiency, have shown higher risks of colonization [3]. HIS is rarely symptomatic. Most cases of HIS have been found incidentally during screening colonoscopies with only a few cases presenting with watery diarrhea and abdominal pain. Another rare presentation reported was HIS presenting with colonic ulceration and acute appendicitis [4-6].

## Case presentation

**Figure 1:**
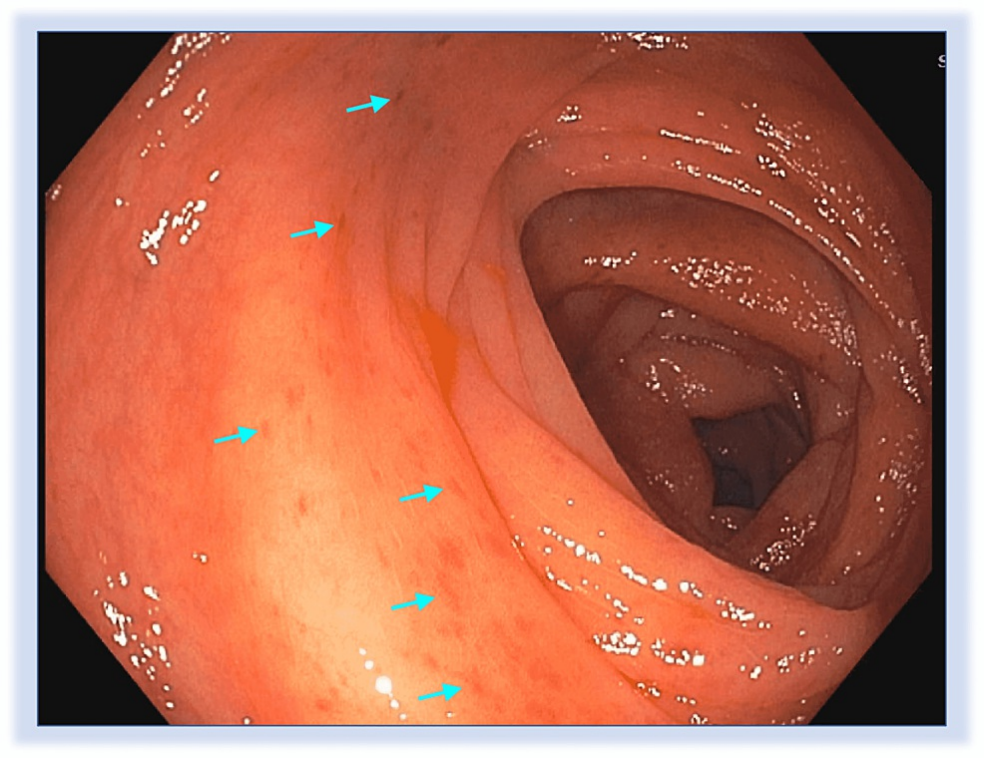
Colonoscopy image Multiple small, 2-3 millimeters, superficial, erythematous lesions on the ascending colon (marked with cyan arrows) are visible.

This is a case of a 57-year-old bisexual, African American male that works as a food preparation worker that presented for an elective, outpatient colonoscopy for further evaluation of hematochezia. He only has a past medical history of HIV on antiretroviral therapy (ART) with an undetected viral load and a CD4 count of 1,401 cells per microliter. In his routine HIV clinic follow-up, he reported a four-month history of intermittent bloating and frequent hematochezia episodes with sometimes passage of blood alone. These symptoms, in addition to a history of a polypectomy of a single, smooth, sessile polyp during a screening colonoscopy when he was 50 years old, prompted the referral to the gastroenterology center for further evaluation. Laboratories obtained within one week of the planned colonoscopy showed a white blood cell count of 9.3 thousand/millimeter^3^ with an absolute neutrophil count of 4.4 thousand/millimeter^3^, and an absolute lymphocyte count of 4.3 thousand/millimeter^3^, hemoglobin of 11.8 grams/deciliter, and a Rapid Plasma Reagin (RPR) that was non-reactive. The colonoscopy was performed and revealed a few small, 2-3 mm, superficial, erythematous lesions on the ascending colon (Figure 1) that were biopsied. No other noticeable findings were seen during the colonoscopy. 

**Figure 2:**
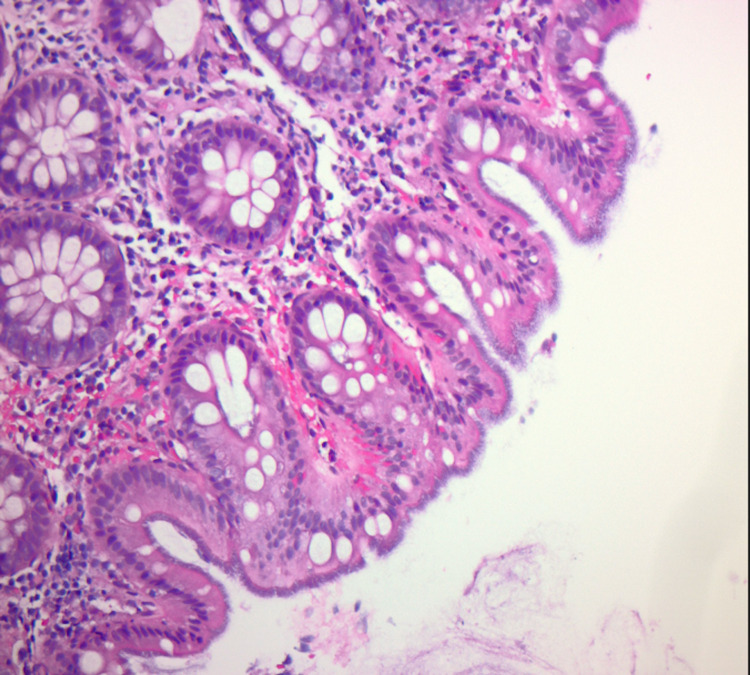
The colonic mucosa has a prominent basophilic, “fuzzy”-appearing brush border at the surface 200X, Hematoxylin and Eosin (H&E) stain.

**Figure 3:**
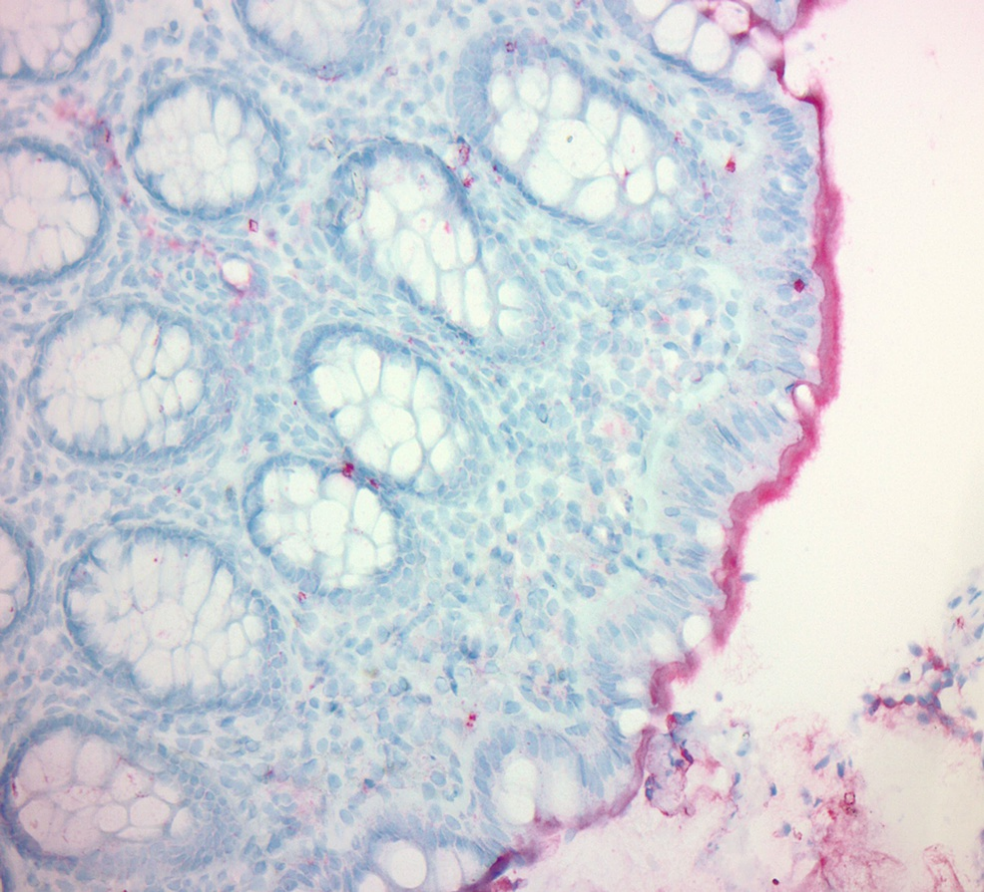
The image highlights spirochetes attached to the surface in red 200X, Spirochete immunostain

The biopsy results showed a prominent basophilic colonic mucosa with a “fuzzy”-appearing brush border (Figure 2) and spirochetes attached to the surface shown in red (Figure 3) consistent with HIS. The patient was prescribed a course of metronidazole of 500 milligrams four times a day for ten days. After completion of therapy, the patient reported resolution of his presenting symptoms.

## Discussion

Currently, it is still unclear if HIS constitutes an actual disease or a commensal colonizer as it has been found in healthy individuals as well as in those that are HIV positive regardless of the degree of immunosuppression. Furthermore, the presence of spirochetes in the stool does not necessarily correlate with HIS or its clinical symptoms. A higher risk for developing intestinal colonization has been reported in men who have sex with men [7] given the change in their rectal microbiota due to the high prevalence of other intestinal infections and frequent antibiotic usage; as well as, in HIV-positive men regardless of sexual orientation or degree of immunodeficiency.

In our case, we can observe a rare presentation of HIS with a four-month bloating sensation and hematochezia. Hematochezia is a symptom that is mostly associated with bleeding diverticulum, arteriovenous malformation, malignancies, neoplastic polyps, inflammatory conditions, and hemorrhoids [8]; rarely associated with HIS. The lack of specific symptoms that can define this disease and the vast degree of severity of the symptoms makes the diagnosis through clinical presentation more challenging [1]. Yet, despite the lack of clear symptoms, it is still important to include this diagnosis in working differentials as there have been multiple reports of spirochetemia and multiple organ failure secondary to bacterial translocation of HIS in critically-ill patients with associated impaired circulation or impaired immune system [9-12]. The presenting symptoms usually seen with HIS have been associated with minimal intestinal invasion, but are not always noticed in patients with spirochetes in the stool. It has been theorized that the mechanism of intestinal invasion, supported by histologic findings of spirochetes adhered to the colorectal epithelium, isn’t noticed in all patients. The reason for this could be that the nature of bacterial translocation is not a continuous one but more of a short-term process; hence, the variations of intestinal invasion [13]. This is why HIS is usually diagnosed during screening colonoscopies of asymptomatic patients as the short-term local variation of the bacterial translocation prevents a homogeneous clinical presentation of HIS.

It's also important to distinguish between HIS and other pathologic yet non-intestinal spirochetes (NIS), like *Treponema pallidum*, famously known to cause syphilis. Unlike NIS, the treatment for HIS is based on clinical presentation, the severity of the symptoms, and the patient’s immune status. Asymptomatic immunocompetent patients with confirmed HIS can be clinically followed while symptomatic and immunocompromised patients should be treated with a ten-day course of metronidazole 500 milligrams four times daily to eradicate the infection [14]. There is currently no data for the need to document the eradication of HIS via repeat colonoscopy.

## Conclusions

Regardless of the degree of immunosuppression, HIS should be a diagnosis considered in all HIV patients with abdominal discomfort and hematochezia, specifically in men who have sex with men. This is an important disease to consider and properly diagnose as effective treatment is available with complete resolution of presenting symptoms. Metronidazole 500 milligrams four times a day for ten consecutive days is currently the treatment for this condition. After proper completion of the antibiotic treatment, there is no current recommendation to repeat the colonoscopy to assess the resolution of HIS. 
